# Classification of Aggregates Using Multispectral Two-Dimensional Angular Light Scattering Simulations

**DOI:** 10.3390/molecules27196695

**Published:** 2022-10-08

**Authors:** Jaeda M. Mendoza, Kenzie Chen, Sequoyah Walters, Emily Shipley, Kevin B. Aptowicz, Stephen Holler

**Affiliations:** 1 Department of Physics and Engineering Physics, Fordham University, Bronx, NY 10458, USA; 2 Department of Physics and Engineering, West Chester University, West Chester, PA 19383, USA

**Keywords:** light scattering, aggregates, T-matrix, machine learning, classification, clusters

## Abstract

Airborne particulate matter plays an important role in climate change and health impacts, and is generally irregularly shaped and/or forms agglomerates. These particles may be characterized through their light scattering signals. Two-dimensional angular scattering from such particles produce a speckle pattern that is influenced by their morphology (shape and material composition). In what follows, we revisit morphological descriptors obtained from computationally generated light scattering patterns from aggregates of spherical particles. These descriptors are used as inputs to a multivariate statistical algorithm and then classified via supervised machine learning algorithms. The classification results show improved accuracy over previous efforts and demonstrate the utility of the proposed morphological descriptors.

## 1. Introduction

From climate change [1] to health impacts [2], the effects of airborne particulate matter are ubiquitous. The optical characteristics of such aerosols are important for understanding their influence in, for example, climate models, or for use in the detection and characterization of unknown respirable particulates [3,4] such as biological threats. The vast majority of such particles are irregularly shaped and often comprised of aggregates of smaller constituent particles which makes detection and characterization particularly onerous [5,6,7,8]. For smooth spherical particles Mie theory allows one to rapidly fit light scattering data to infer morphological information such as size and composition [9], however, for aggregates this is not so straightforward. The aggregation of small particles to form a larger cluster results in an irregular boundary and light scattered from such a cluster undergoes multiple scattering events and interference that lead to a complicated speckle pattern. Furthermore, the spherical symmetry is lost in aggregates resulting in a scattering pattern that varies azimuthally so that even simple one-dimensional scattering slices are not useful in developing a clear picture of the effect of aggregate scattering.

Two-dimensional Angular Optical Scattering (TAOS) is an experimental approach that looks at a large solid angle and records the speckle pattern from light scattered by airborne particles [10,11]. Variations in the light collection geometry have resulted in nearly 2π steradians each in both the forward and backward scattering signal; more typical approaches capture a significantly smaller solid angle of scattering [3,4,10,12,13,14]. The TAOS patterns are typically recorded with a CCD camera, and, in some instances, multiple light sources have been employed to record patterns at different wavelengths in a single shot [5,15]. This has the advantage of being able to capture dispersion information so that not only information about the shape of the aggregate can be discerned but so too can the material composition (i.e., refractive index).

Previous studies have shown that particles may be classified according to their TAOS patterns and that these classifications generally fall in line with the known aggregate morphology. We have used multispectral TAOS to enhance classification efforts using morphological descriptors that are related to and/or evoke physical characteristics of the cluster. [15] These experimental results proved promising and suggest that with the chosen descriptors, classification of unknown aerosols based on TAOS pattern data was at least as good as earlier efforts that did not use these morphological descriptors [16,17,18]. The current work expands on this by controlling for experimental variation (e.g., cluster size, position in the beam, laser intensity fluctuations, etc.) by employing two-dimensional multispectral light scattering calculations from fixed aggregate geometries and different materials, and seeks to demonstrate that improved classification accuracy using these physically-relevant descriptors. Multivariate statistical analyses are performed on the morphological descriptors obtained from TAOS calculations and the results are then input into different machine learning classification routines. The results show improvements over previous efforts indicating that the challenge of accurately assigning aerosols to a known class according to their TAOS patterns is not insurmountable.

In what follows we describe our approach including cluster generation and light scattering calculations. This is followed by a description of the morphological descriptors and the determination of them for the simulated aggregates. The multivariate statistical approach is presented along with the classification routines, and finally the resulting classifications are shown.

## 2. Simulations and Descriptors

### 2.1. Multiple Sphere T-Matrix Code and Aggregate Generation

TAOS patterns from aggregates were computed using Multiple Sphere T-Matrix (MSTM) code developed by Dan Mackowski and Mike Mishchenko [19,20]. The MSTM code was developed in Fortran-90 for computing the electromagnetic scattering and absorption properties from arbitrary arrangements of spherical particles subject to the constraint that the particle surfaces not intersect each other. A position file was constructed to specify the location and radius of the spherical particles that comprise our aggregates, and served as the input for the MSTM code.

In order to determine the locations used in the position file we started with the nominal diameter of the cluster we wished to consider. This volume was filled with random points that represent the location of the constituent spheres. The size of the constituent sphere was then set. This results in a structure of overlapping and intersecting spheres. Spheres were then removed from the volume so that no overlapping (intersecting) volumes remained. This yields a loosely packed, disjoint cluster of randomly positioned spheres, so in order to form a physically realizable cluster, the radii of the remaining constituent spheres were adjusted until they touched their nearest neighbor. This resulted in a tight-packed, nearly spherical cluster configuration in which the polydispersity of the constituent spheres was small. We computed the Coefficient of Variance (CV), defined as the standard deviation of the constituent particle size divided by the mean constituent particle size. For the four hundred aggregates generated we compute a mean CV of 5.4%.

**Figure 1:**
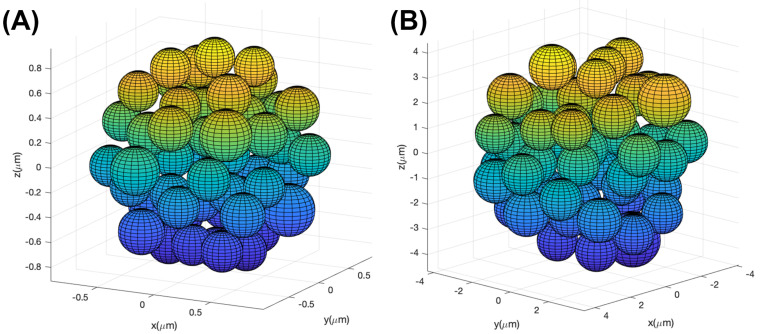
Representative aggregate framework for a (**A**) 2 μm and a (**B**) 10 μm overall cluster diameter. The 2 μm diameter aggregate is comprised of constituent spherical particles having a diameter of 0.38 μm while the spherical particles that make up the 10 μm aggregate are 1.9 μm in diameter. These are simply examples of cluster morphology and different frameworks were created that were comprised of different constituent particle sizes.

Aggregare frameworks were created for overall cluster diameters of 2 μm, 4 μm, 6 μm, 8 μm, and 10 μm. Constituent particles varied depending on the overall aggregate size but ranged from 0.38 μm to 5 μm. For each of the constituent sphere diameters, twenty unique aggregate frameworks were created. Figure 1 shows examples of frameworks for two different cluster configurations. Figure 1A shows a 2 μm overall cluster diameter comprised on 0.38 μm diameter spherical particles while Figure 1B is a 10 μm overall cluster diameter made up of 1.9 μm diameter spherical particles. These are merely examples and other aggregate frameworks having the same overall diameter were comprised of different constituent particle sizes.

The position files were input into the MSTM code along with the complex refractive index (i.e., material) of the constituent particles. The same position frameworks were used for each of the different materials so that for any given configuration there were nine different calculations at each of the incident wavelengths. Far field, angularly resolved scattering calculations were performed over the full 4π sr solid angle at 405 nm, 532 nm, and 660 nm. This spread of wavelength across the visible spectrum allowed us to consider the effects of dispersion in the calculation and classification. For each aggregate framework and each incident wavelength scattering patterns were computed for each of nine different materials with varying levels of absorption. Table 1 shows the materials and the complex refractive indices (m = n + *i*κ) used as inputs to the MSTM code.

### 2.2. Morphological Descriptors

For analysis and classification the speckle data, the two-dimensional angular optical scattering patterns was first partitioned into forward and backward scattering hemispheres. Rather than use the full hemisphere of data, we selected a subimage, the extent which was chosen to mimic the solid angle subtended by a camera used to capture TAOS data in actual experimental configurations. To maximize the amount of data we considered four different subimages in the scattering hemispheres; this effectively quadrupled the data available. This is justified by considering that a rotation of the particle would, for the circularly polarized illumination used, result in a corresponding rotation of the TAOS pattern.

**Figure 2:**
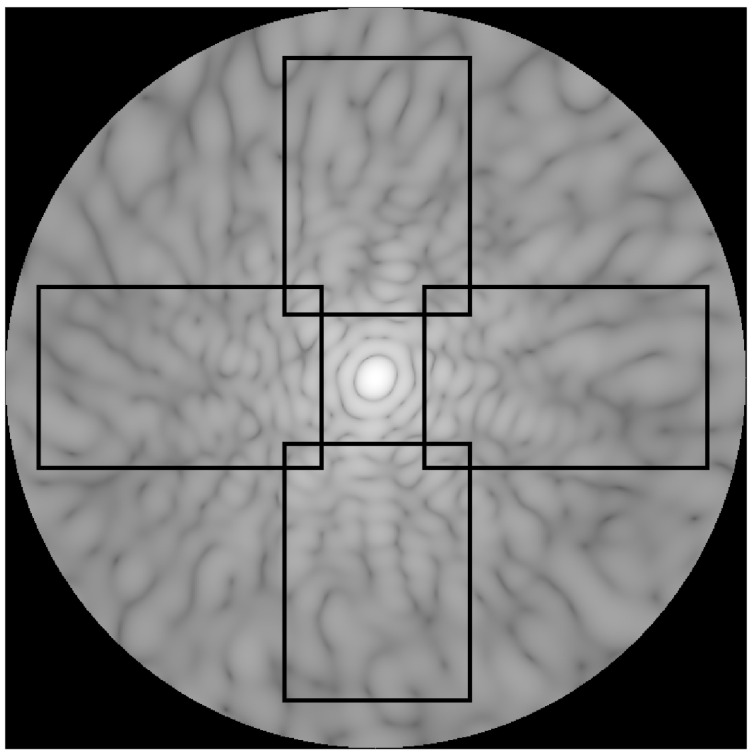
Image of the forward scattering hemisphere showing four regions from which subimage panels were formed. These panels represent regions observed by cameras in experimental work, and provide a good sample of the scattering image. The use of four panels quadrupled the amount of data that was analyzed.

Figure 2 shows the arrangement of the panels in a scattering hemisphere. Each of these panels was processed to reduce the information contained into morphological descriptors that were used for analysis and classification. We sought to determine useful parameters that would both represent salient characteristics of the TAOS patterns and elicit imagery reflective of the physical characteristics of the aggregates. The six morphological descriptors that were employed are:

1. Mean Intensity;
2. Image Entropy;
3. Modified Autocorrelation;
4. Fractal Dimension;
5. Speckle Width;
6. Modulation Depth.

These six morphological descriptors were computed for each of the wavelengths used in the MSTM simulations resulting in 18 data points per aggregate. This descriptor data was then compiled into a matrix for input into a Principal Component Analysis (PCA) algorithm, the results of which subsequently were input into different machine learning classification algorithms.

#### 2.2.1. Mean Intensity

The first descriptor we considered was the mean intensity of the image. The mean intensity of the sub-images extracted in the four panels from the scattering hemisphere was computed for each of the scattering patterns associated with the aggregate framework. We associate the mean intensity of the image with the overall cluster size.

#### 2.2.2. Image Entropy

In image processing, entropy (*S*) is a measure of the randomness of the image. The entropy value can be used to characterize the texture of an image. The entropy is computed by first computing a normalized histogram (*p*) of the counts of the image. Once the histogram of the image counts is determined, the entropy is computed by applying Equation (1):

(1) $$S=-\sum_{i}p_{i}log_{2}\left(p_{i}\right),$$

where $p_{i}$ is the *i*th bin of the histogram. In effect, $p_{i}$ is the probability that an intensity value occurs in a particular bin in the histogram. Because of its relation to image texture and randomness we associate the image entropy with the texture of the aggregate.

#### 2.2.3. Modified Autocorrelation

The modified autocorrelation is a cross-correlation of the scattering panel segment with its flipped self. The panel segment is selected to be centered on a plane that bisects the aggregate. In conventional scattering calculations this plane corresponds to the ϕ = 0${}^{\circ }$ angular direction. The panel segment is flipped about this plane to produce an inverted image and then the correlation coefficient (*r*) is computed according to Equation (2).

(2) $$r=\frac{\sum_{m}\sum_{n}\left(A_{mn}-\bar{A}\right)\left(A_{mn}^{f}-\bar{A}\right)}{\sum_{m}\sum_{n}{\left(A_{mn}-\bar{A}\right)}^{2}}$$

where *A* is the image panel, *A${}^{f}$* is the flipped image, and $\bar{A}$ is the mean intensity of the image panel. This descriptor was chosen because it informs about the symmetry of the aggregate. A perfect spherical scatterer would produce rings which, when flipped, reproduce the same pattern and therefore have an *r* value of unity. As the cluster loses this symmetry the *r* will decrease.

#### 2.2.4. Fractal Dimension

The fractal dimension of the scattering pattern is computed to look for features that would reflect the arrangement of the constituent spheres within the aggregate. Aggregates, particularly soot chains, have a long history of being characterized by their fractal dimension [21,22,23]. A two-dimensional box counting algorithm was implemented on the panel images of the scattering [24]. Prior to implementing the box count, the image was binarized based on the mean value of the image.

The non-zero regions of the binarized scattering image is covered with boxes of size *R* and the number of required boxes (*N*) is then counted. The box counting fractal dimension ($D_{f}$), or Kolmogorov dimension [24], is readily determined because the number of boxes required to cover the space is

(3) $$N=R^{-D_{f}}$$

The sizes of the boxes are powers of two and, if necessary, the image is padded with zeroes to obtain an image of 2${}^{p}$. The box counting algorithm then computed the *N* for all values from 1 to *p*. The fractal dimension is then determined from the slope of the line given by Equation (3). The fractal dimension is expressed as

(4) $$D_{f}=-\frac{\Delta ln(N)}{\Delta ln(R)}.$$

#### 2.2.5. Speckle Width

The speckle width is another parameter that is related to the overall size of the aggregate [25]. This metric has been used previously for characterizing clusters based on their light scattering patterns. The speckle width is computed by performing a two-dimensional autocorrelation on the scattering panel subimage. A threshold of 50% of the peak value of this autocorrelation is used to produced a binarized result. The result is a central peak that has an elliptical shape. The major axis of this ellipse is determined, in pixels, which was subsequently converted to an angular measure (Δθ) using the known scaling factor. This value represents the nominal width of the speckle features in the scattering image.

#### 2.2.6. Modulation Depth

Slices (one dimensional scattering cuts) were taken in the “equatorial” plane of the segmented image and the depth of modulation was computed between peaks and troughs. The depth of modulation is expected to vary with the imaginary part of the refractive index.

### 2.3. Classification

Machine learning systems are classified by the type of supervision they receive during training. Classification is a type of supervised learning in which the algorithm makes predictions on the dataset where every instance has an expected output. The multilayer perceptron (MLP) classifier, k-nearest neighbors (KNN) classifier, random forest classifier, and extra trees classifier were the supervised learning algorithms examined for classifying aerosol aggregates based on either cluster size or material composition.

The forward scattering and backward scattering datasets were the feature values. The cluster size and material composition were the labels. Since the data was slightly imbalanced and this could result in misclassification, 2490 samples per cluster size and 1520 samples per material composition were randomly selected.

#### 2.3.1. Feature Scaling

Depending on the machine learning algorithm, feature scaling enhances the algorithm by transforming input values to have the same scale. Whereas MLP and KNN require data to be scaled because they are sensitive to ranges of feature values, random forest and extra trees do not require feature scaling because the splitting of a node does not depend on the feature’s scale [26].

#### 2.3.2. Model Selection: Cross-Validation

Repeated *k*-fold is a cross-validation technique used for model selection by evaluating the model’s performance. It randomly samples and shuffles the data by splitting the data into *k*-folds. Training occurs on the k−1 folds; the remaining folds are used to assess the model’s performance. The *k*-fold process was repeated five times. The mean performance score and the standard deviations were used to select the appropriate models for the four scenarios: backward scattering and cluster size, backward scattering and material composition, forward scattering and cluster size, and forward scattering and material composition. The MLP classifier was the selected model to train because it consistently outperformed the other models.

The data was split into training and test sets to determine how well the machine learning algorithm performs and to prevent overfitting or underfitting. The training set contained 70% of the original dataset, and the test set included 30% of the original dataset. The training set was used to fit the MLP classifier, and the test set was used to evaluate the model’s fit.

#### 2.3.3. Hyperparameter Tuning

Scikit-Learn’s GridSearchCV() is a hyperparameter optimization function to improve the model’s performance through cross-validation. It searches for the optimal combination of parameters for the model by iterating through them and fitting the training set using each combination. Repeated *k*-fold was the selected method to address the overfitting and underfitting of the test set by assessing the validation set and then evaluating the test set [26].

#### 2.3.4. Classifiers

##### Multilayer Perceptron

MLP is a type of artificial neural network. It uses a multilayer perceptron algorithm and trains iteratively through back-propagation by computing the gradients. The multilayer perceptron has an input layer, one or more hidden layers, and an output layer. The algorithm goes through the training set several times. During each pass, the input layer was the set of neurons of the scattering data. The values in the hidden layers were transformed from the input layer using a weight linear summation, then a rectified linear unit activation function. The output values of the labels were the transformed values from the last hidden layer received by the output layer. The algorithm used the cross-entropy loss function to measure the error between the expected output and the network’s actual output. Then the algorithm passed through each layer in the reverse direction until it reached the input layer to compute the amount of error contributed from each connection and adjusts the connection weights to decrease the error [26].

##### K-Nearest Neighbors

K-Nearest Neighbors (KNN) Classifier uses a Euclidean distance metric to evaluate feature similarity. The algorithm measured the distance between the data points of the training samples from the forward or backward scattering dataset. The cluster size or material composition was predicted based on the k-nearest neighbors of each data point with the highest vote counts. The optimal number of neighbors, the *k* value in k-nearest neighbors, was tuned through GridSearchCV [26].

##### Random Forest

Random Forest (RF) Classifier is an ensemble learning method. It fits a group of decision trees on random subsets with the replacement of the training set. A decision tree makes a prediction using the root node and leaf node. Different numbers of trees are constructed as a result of hyperparameter tuning (Section 2.3.3) using GridSearchCV. The root node was the training set containing only the forward or backward scattering dataset that produced the optimal split with the purest training instances. The dataset was split by randomly selecting different forward or backward scattering values and measuring the Gini impurity. Then, the sequences reached the leaf nodes and further segregated the dataset until the leaf nodes could not be divided anymore. Individual trees returned their predicted labels, and the model’s prediction was the one with the most votes [26].

##### Extra Trees

Extra Trees Classifier is also an ensemble learning method, similar to the random forest classifier. However, when constructing the decision trees, random thresholds for the features were used instead of the optimal split point [26].

## 3. Results and Discussion

There are three lenses through which one can see distinctions of the TAOS patterns among the different aggregates. The TAOS patterns provide a high-level view with coarse level of distinction with a minimal ability to discriminate among particles. Principal Components Analysis provides the first layer of multivariate statistical analysis and begins to extract details using the morphological descriptors that allows for greater discrimination. The classification algorithms applied, with the scores values obtained from the Principal Component Analysis as inputs, demonstrate the ability to assign aggregates at a higher rate than previously performed [15,17].

### 3.1. Scattering Results

Morphologically complex aerosols, that is, aggregates comprised of spherical or other shaped constituent particles forming close-packed arrangements, when illuminated by light, produce a speckle pattern resulting from interference and multiple scattering events. This speckle pattern contains information about the size and shape of the aggregate, and the material from which it is comprised. The most obvious features are the size of the individual speckles, which have an inverse relationship with overall aggregate size; large clusters produce a speckle pattern with smaller islands of intensity. Consequently, the density of the features increases with aggregate size, both geometric and optical. The optical size is defined as the ratio of the aggregate circumference to the illuminating wavelength so decreasing the illuminating wavelength results in a larger particle as seen by the light. Consequently, the speckle observed in TAOS patterns will be more dense with smaller speckle features at shorter wavelengths.

**Figure 3:**
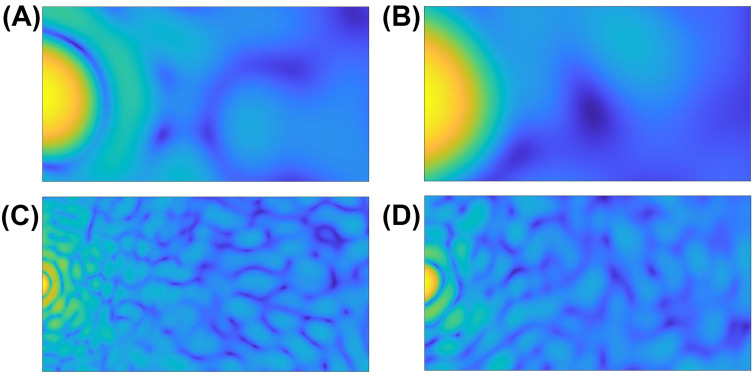
Segments of forward scattering TAOS data showing effect of both geometric and optical size of the aggregate. (**A**) 2 μm nominal aggregate diameter comprised of 0.5 μm diameter spheres illuminated at 405 nm; (**B**) 2 μm nominal aggregate diameter comprised of 0.5 μm diameter spheres illuminated at 660 nm; (**C**) 10 μm nominal aggregate diameter comprised of 2.5 μm diameter spheres illuminated at 405 nm; (**D**) 10 μm nominal aggregate diameter comprised of 2.5 μm diameter spheres illuminated at 660 nm.

Figure 3 shows representative segments used for the feature extraction from two different clusters sizes (2 μm and 10 μm) at two different wavelengths (405 nm and 660 nm). Panels (A) & (B) are the smaller aggregate while (C) & (D) are from the larger one. The left column (A & C) is 405 nm illumination while the right (B & D) is 660 nm illumination. The features of the 532 nm data (not shown) lie, as expected, between the two shown.

As mentioned earlier, these speckle features were used to formulate morphological descriptors, characteristics of the two-dimensional scattering pattern that are dependent on the size and composition of the aggregate and may be used with classification algorithms to separate classes of particles. The simulated scattering patterns were computed at three different wavelengths to provide information about optical size and to capture the effects dispersion due to the aggregate material. The six different morphological descriptors at the different wavelengths yielded 18 values for input to the multivariate and classification algorithms.

### 3.2. Principal Component Analysis

**Figure 4:**
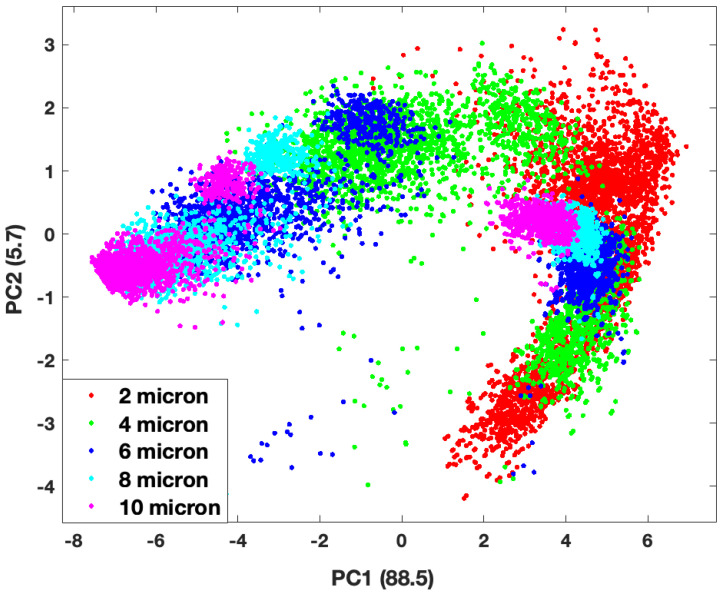
Scatter plot of the scores values for first 2 principal components of the forward scattering dataset by cluster size.

**Figure 5:**
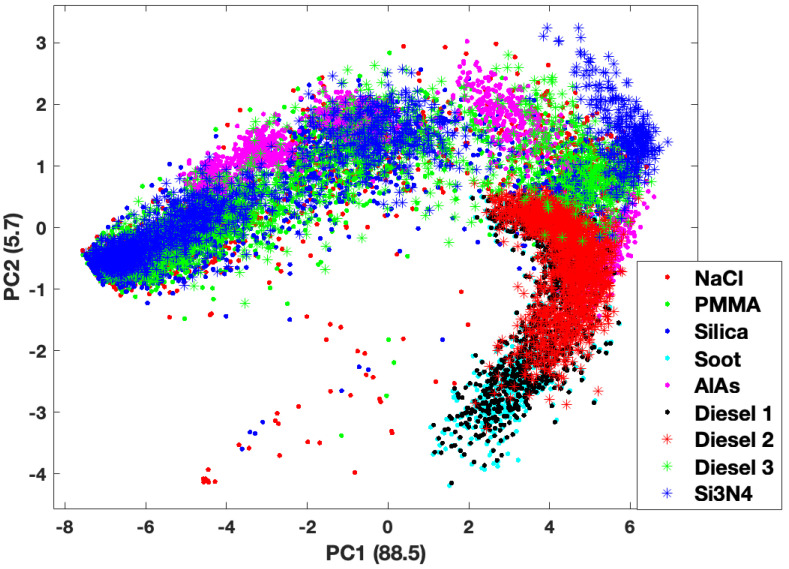
Scatter plot of the scores values for first 2 principal components of the forward scattering dataset by aggregate material.

Principal Component Analysis (PCA) was performed on the matrix of descriptors (13,964 × 18) to recast the data in a way that would elucidate information about and provide separation among the classes (e.g., cluster size, material composition). The first two Principal Components (PCs) constitute 94% of the information contained in the forward scattering data set. Figure 4 and Figure 5 show scatter plots of the PC Scores of the forward scattering data set denoting cluster size and material composition, respectively. Grouping is noted but there is significant overlap of groups (either size or material) in these projections.

**Figure 6:**
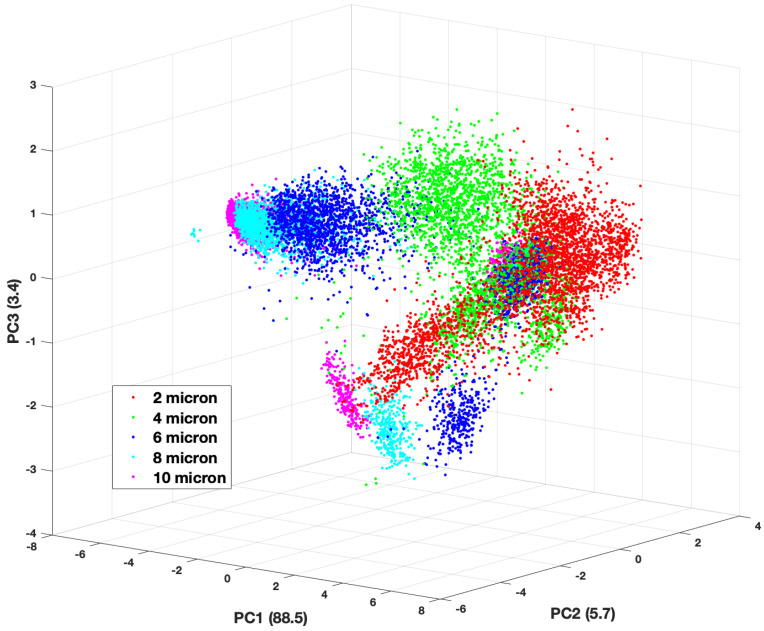
Three-dimensional plot of the scores values for first 3 principal components of the forward scattering dataset by cluster size.

**Figure 7:**
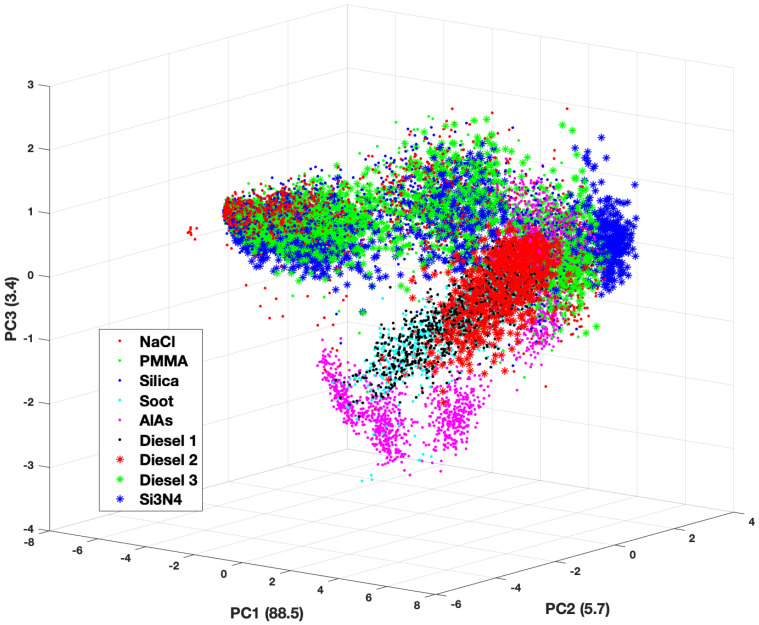
Three-dimensional plot of the scores values for first 3 principal components of the forward scattering dataset by cluster material.

The third PC only contains about 3% of the information content in the forward scattering data set, however, this is enough to introduce more separation among the groups. While there is still some overlap of the groups, it is much easier to observe separations among them in the three-dimensional plots, especially when it comes to cluster size. Figure 6 and Figure 7 show three-dimensional plots of the first 3 PCs, which constitute 97% of the information in the forward scattering data set. Figure 6 shows a scatter plot of the Score values of the first three PCs with the overall cluster size distinguished, while Figure 7 shows the same plot according to the material (refractive index) of the cluster. There are clear separations among the data points by cluster size. This is not unexpected as overall aggregate size plays a significant role in the the density and size of the speckle features seen in the TAOS patterns.

Examining the data from the material perspective requires a closer look at the materials involved. Nine materials were chosen with varying amounts of absorption in the complex refractive index. The band that extends down and to the left in the figures is comprised of those materials that have a relatively strong component to the imaginary part of the refractive index (i.e., high absorption) across all three wavelengths. The weakly absorbing or completely real refractive index materials comprise the rest of the data. Within these two branches one can see how the overall cluster size affects the separation of the groups.

The backward scattering data produces similar plots showing clear aggregation of the PC scores as a function of overall cluster size. As with the forward scattering data, the separation by material is observable but not nearly as distinct. The visual separation of the classes in best validated and quantified with the machine learning classification routines.

### 3.3. Classification

**Figure 8:**
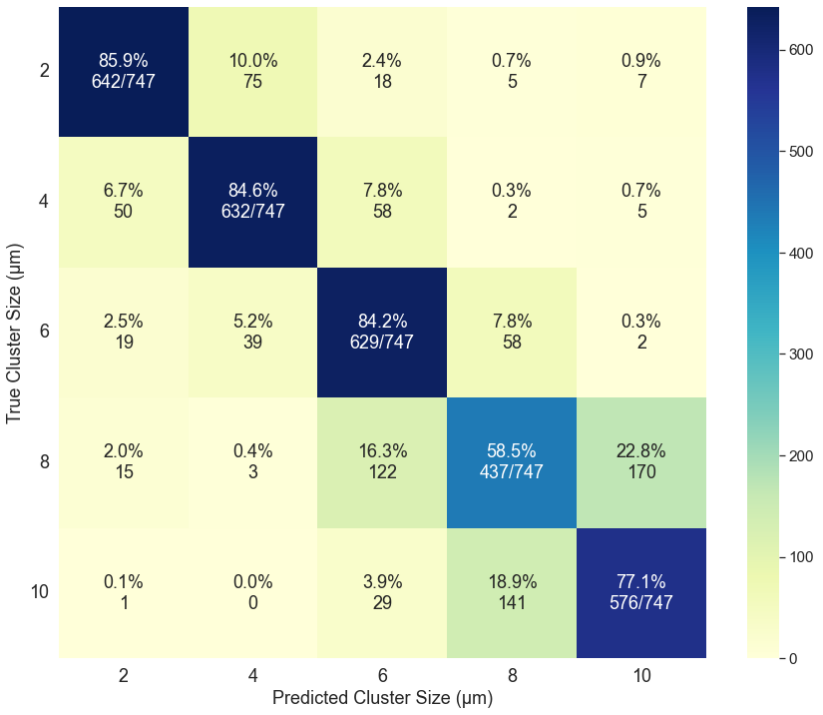
Confusion matrix for the classification based on cluster size using the forward scattering data for the analysis.

**Figure 9:**
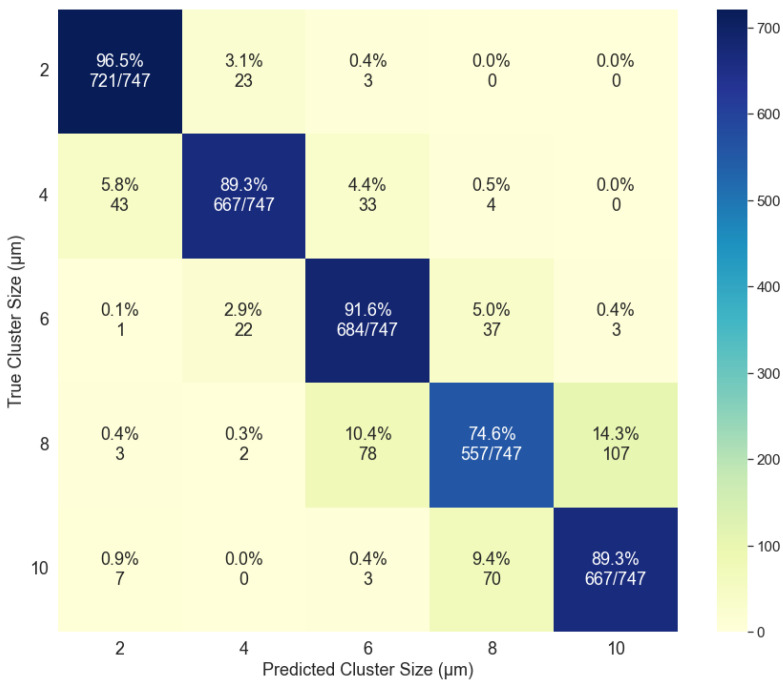
Confusion matrix for the classification based on cluster size using the backward scattering data for the analysis.

The MLP classifier performed the best during the cross-validation process (Section 2.3.2). The test data comprised 30% of the sample set corresponding to 747 images for each cluster size. The accuracy of the model was dependent on the data being classified: (1) cluster size forward scattering (2) cluster size backward scattering, (3) material composition forward scattering, and (4) material composition backward scattering. The accuracies were determined for each case to be: (1) 78.1%, (2) 88.3%, (3) 47.4%, and (4) 55.0%. The results were compiled and a confusion matrix was created for each condition. The confusion matrices for the cluster size classification are shown in Figure 8 and Figure 9 and illustrate good classification results. For the data based on the cluster size in the forward scattering direction (Figure 8), the average specificity was determined to be 94.5% while the average sensitivity was found to be 78.1%. The same analysis for the backward scattering data (Figure 9) yields 97.1% for the specificity and 88.3% for the sensitivity.

## 4. Conclusions

Two-dimensional light scattering signals from complex aggregates are comprised of speckle patterns that are sensitive to morphological characteristics of the cluster. These characteristics imprint themselves on the TAOS signal and can be used to discriminate among different classes of clusters.

The morphological descriptors presented, when used as inputs for the multivariate analysis and machine learning algorithms, are inline with recent experimental results [15], and provide improved classification over previous classification attempts [27]. The current work demonstrates an accuracy of 88% for the backward scattering analysis compared to 74% in our previous work. No similar comparison with experimental results could be made with the forward scattering TAOS data but the present work demonstrates good classification accuracy in this case as well.

The classification based on material composition proved more challenging. The results demonstrated about 50% effectiveness but this is not surprising given that the scattering is dominated by structural effects. Material composition produces a greater influence in the backward direction and this is reflected in the greater classification accuracy among the backscattering data set.

Light scattering continues to be an important tool for characterizing and understanding the optical properties of complex non-spherical aerosol particles. Overall, the morphological descriptors presented and the classification algorithms employed demonstrate improvements over previous results for a challenging task. The approach presented provides additional tools for the analysis of TAOS data.

## Figures and Tables

**Table 1:** List of the materials and corresponding complex refractive indices used as inputs with the position files for the MSTM code.

	405 nm		532 nm		660 nm	
Material	n	κ	n	κ	n	κ
Aluminum Arsenide	3.8210	0.10884	3.2695	4.097 × 10${}^{-3}$	3.1009	6.9847 × 10${}^{-4}$
Diesel Soot	1.5308	0.2505	1.6044	0.2762	1.6520	0.2890
Diesel Soot (mod 1)	1.5308	0.23297	1.6044	0.25687	1.6520	0.26877
Diesel Soot (mod 2)	1.5308	0.08768	1.6044	0.09667	1.6520	0.10115
Diesel Soot (mod 3)	1.5308	2.51 × 10${}^{-3}$	1.6044	2.76 $\times 10^{-3}$	1.6520	2.89 × 10${}^{-3}$
PMMA	1.5051	0	1.4934	0	1.4880	0
Silica	1.4696	0	1.4607	0	1.4563	0
Silicon Nitride	1.9640	2.425 × 10${}^{-3}$	1.9206	0	1.9020	0
Sodium Chloride	1.5810	0	1.5508	0	1.5420	0

## Data Availability

Data are available upon request.

## Abbreviations

The following abbreviations are used in this manuscript:

KNN	K-Nearest Neighbors
MLP	Multilayer Perceptron
MSTM	Multiple Sphere T-Matrix
PC	Principal Component
PCA	Principal Component Analysis
RF	Random Forest
TAOS	Two-dimensional Angular Optical Scattering
